# Relationship between emotional intelligence and learning motivation among college students during the COVID-19 pandemic: A serial mediation model

**DOI:** 10.3389/fpsyg.2023.1109569

**Published:** 2023-03-15

**Authors:** Yuxi Tang, Weiguang He

**Affiliations:** College of Social Sciences, Shenzhen University, Shenzhen, China

**Keywords:** COVID-19 pandemic, emotional intelligence, learning motivation, self-efficacy, social support

## Abstract

The vital influence of emotional intelligence on college students’ learning motivation has received considerable attention. This study analyzed not only the relationship between emotional intelligence and college students’ learning motivation during the COVID-19 pandemic, but also the serial mediating roles that self-efficacy and social support play in this relationship. Using a cross-sectional survey design, we collected data from 336 college students across 30 provinces in China, using four well-established scales measuring emotional intelligence, learning motivation, self-efficacy, and social support. We analyzed the mediating effects using the Bootstrap method. The results showed that emotional intelligence positively predicted learning motivation, and that self-efficacy and social support played serial mediating roles between emotional intelligence and learning motivation. This finding suggests the need for interventions to help college students develop emotional intelligence during the COVID-19 pandemic, and that fostering college students’ self-efficacy and providing multiple social supports would help improve their motivation and academic performance.

## Introduction

The coronavirus disease 2019 (COVID-19) continues to erode people’s psychosomatic wellbeing and seriously affect students’ lives and work in many countries because of the continuous mutation and spread of the virus (ElTohamy et al., 2022; Li, 2022). There is widespread interest and concern about the high levels of anxiety and depression experienced by university students worldwide during the COVID-19 pandemic (Zhan et al., 2021; Reid et al., 2022) and the difficulties they face in their studies (Telli et al., 2021; Curcio et al., 2022; Shi et al., 2022). During the COVID-19 pandemic, college students are likely to face challenges of psychological stress (Husky et al., 2020; Dumitrache et al., 2021) and academic distress (Wood et al., 2022)，and it is significant to find protective interventions for mental health and learning. Dealing with stress and coping with challenges from multiple sources often requires college students to have better emotional regulation abilities (Molina, 2022).

Emotional intelligence, which has the potential for mental health protection, has received increased attention in recent years (Mancini et al., 2022). The positive impact of emotional intelligence on education has also received widespread attention from practitioners around the world (Parker et al., 2006; Pishghadam et al., 2022). Emotional intelligence is the mental ability of individuals to perceive, understand, and assess their own and others’ emotions, as well as to manage, regulate, and apply them (Salovey and Mayer, 1990; Salovey and Grewal, 2005; Mayer et al., 2016), and is considered a positive factor for psychological well-being (Schutte et al., 2007). The significance of emotional intelligence for the maintenance of college students’ learning is heightened during the COVID-19 pandemic (Guillen et al., 2022). In addition to recognizing the importance of emotional intelligence, there is a growing need to explore the mechanisms through which emotional intelligence positively influences college students’ learning.

This pandemic has profoundly affected students’ learning (Pokhrel and Chhetri, 2021), and many college students have had to hastily embrace the e-learning approach (Aguilera-Hermida, 2020; Gonzalez-Ramirez et al., 2021). Motivation is an essential factor for college students to stay interested and actively engaged in their learning. In this process college students’ learning motivation has been impacted (Daniels et al., 2021), and maintaining good learning motivation has become something that many college students strive to do. In particular, college students may be required to undergo online forms of education during the pandemic, forcing the need for increased time spent online, which further exacerbates their risk of problematic internet use (Gavurova et al., 2022) and to some extent distressing learning (Omari et al., 2022).

Learning motivation is very critical for students as a key predictor of learning success (Allen, 1999; Liu et al., 2012; Pelikan et al., 2021). For this reason, it is an interesting question to figure out what are the protective factors of college students’ learning motivation. Students with good emotional intelligence are more likely to have good academic experiences that positively influence their academic motivation. Emotional intelligence may be one of the powerful protective factors of learning motivation (Jaeger and Eagan, 2007; Rauf et al., 2020; Tam et al., 2021), but the mechanisms of the influence of emotional intelligence on learning motivation are unclear, especially in the context of the pandemic, and exploring these mechanisms is the purpose of this research.

Globally, the negative impact of pandemics on college students’ learning is considerable (Connolly and Abdalla, 2022). There is an urgent need to identify the factors that play an influential role in mediating the relationship between emotional intelligence and academic motivation to develop effective interventions accordingly. As emotional intelligence is a promising protective factor for the academic success and mental health of college students, it is important to understand some of the mechanisms that can better leverage emotional intelligence. This may help to develop better interventions to enhance the learning motivation of college students, which would benefit the academic and psychological well-being of many college students around the world.

### The relationships between emotional intelligence and learning motivation

Emotional intelligence has a major function in decreasing worry (Zysberg and Zisberg, 2022), promoting positive mental states (Persich et al., 2021; Mohamed et al., 2022), and helping people to better adapt to challenging social environments. Emotional intelligence also has a positive effect on students’ ability to control their own emotions, recognize the emotions of others, maintain a good state of mind, and take appropriate actions (Tapia, 2001). Recent studies have found a facilitative effect of emotional intelligence on student learning (Alam et al., 2021; Chang and Tsai, 2022; Iqbal et al., 2022). Students’ emotional intelligence can also help them make positive progress in academic and athletic performance (Pishghadam et al., 2022; Mercader-Rubio et al., 2022a).

Learning motivation has been identified as the key psychological ability that motivates students to value learning, focus on learning goals, manage learning strategies, and regulate learning engagement (Vallerand et al., 1992; Vansteenkiste and Lens, 2006; Walker et al., 2006) and involves both autonomous decisions and external influences (Kusurkar et al., 2013; Litalien et al., 2017). Learning motivation can usually be divided into intrinsic and extrinsic motivation. Intrinsic motivation implies that students learn as an end in itself and is often associated with good learning emotions (Carreira, 2011). Extrinsic learning motivation means that learners are motivated to learn by external conditions (Suzuki and Sakurai, 2011). A large body of research has shown that intrinsic motivation contributes to satisfactory educational outcomes (Taylor et al., 2014; Ryan and Deci, 2020), and extrinsic motivation has also been shown to have a positive effect on student performance in some cultural backgrounds (Liu et al., 2020), implying that both extrinsic and intrinsic forms of motivation can have a positive impact on learning (D'Lima et al., 2014; Cheng, 2019; Diseth et al., 2020). Learning motivation is an integral motivator for students’ academic success and athletic performance (Linnenbrink and Pintrich, 2002; Guay et al., 2010; Wulf and Lewthwaite, 2016).

Learning motivation can be understood as an emotional experience in learning (Zhu et al., 2022), and good academic motivation in college students requires positive emotions as a foundation. When college students can identify and control their negative emotions in the face of learning difficulties, they are likely to adopt more effective learning styles and thus experience more positive learning emotions. When a college student is emotionally satisfied, he or she may have a more positive and optimistic attitude toward learning (Ding, 2022). Self-determination theory provides an understanding framework of the factors that promote and undermine motivation, and it suggests that people have a natural tendency to learn and develop when their psychological abilities and needs are well met (Trolian and Jach, 2020; Hosseini et al., 2022). Some college students face greater academic challenges, and these academic challenges may affect their motivation to learn during the pandemic (Ozer and Schwartz, 2020). The higher levels of emotional intelligence are even more valuable in protecting students’ motivation at this time. Previous studies have found that emotional intelligence may be strongly associated with learning motivation (Arias et al., 2022; Mercader-Rubio et al., 2022a), but this relationship needs to be further tested in a broader group of students. It would be valuable to further probe the mechanisms by which emotional intelligence affects learning among college students to better utilize the positive effects of emotional intelligence on education.

### The mediating role of self-efficacy in the relationship between emotional intelligence and learning motivation

Self-efficacy is one’s overall judgment and confidence in one’s ability to behave (Schunk, 1991; Bandura, 2012; Marsh et al., 2019). As an individual’s overall perception of his or her abilities, self-efficacy can have an impact on people’s willingness to adopt behaviors (Bandura, 1977). It influences cognitive development and can motivate people to take positive actions and achieve predetermined goals (Bandura, 1993). Studies have demonstrated the relevance of emotional intelligence and self-efficacy (Salavera et al., 2017; Cong and Li, 2022). Emotional intelligence can help people regulate and control their emotions, help them experience more positive emotions, deal with problems and relationships better, and thus have more positive evaluations of themselves and things around them, which means that people with higher emotional intelligence are likely to have good self-efficacy (Moafian and Ghanizadeh, 2009).

It was found in previous studies that self-efficacy may influence the level of learning motivation of college students and be an important factor in protecting their motivation (Schunk, 1991). Recent studies have found that self-efficacy is also considered a significant determinant of students’ motivation (Zimmerman, 2000; Deng et al., 2022). Self-efficacy, as an enduring intrinsic driving force, makes college students more resilient and perceive more positive emotions when dealing with learning difficulties and challenges during the pandemic (Alemany-Arrebola et al., 2020). This affirmation of self-efficacy may have a positive impact on learning motivation formation (Bong, 2004). Self-efficacy may make it easier for college students to experience a sense of accomplishment in their studies and motivate their willingness to pursue better academic performance (Dogan, 2015), which may have a positive impact on their motivation to learn. Previous research has found a correlation between self-efficacy and both emotional intelligence and learning motivation (Andriani et al., 2022). Self-efficacy and learning motivation may also be closely linked, although whether self-efficacy is a mediating variable in the relationship between emotional intelligence and learning motivation remains unclear. It can be hypothesized that self-efficacy, an important positive psychological resource, may play a mediating role between emotional intelligence and learning motivation during the pandemic.

### The mediating role of social support in the relationship between emotional intelligence and learning motivation

Social support is one’s overall perception of the social relationships in which one is embedded and the resources available from other people in the environment to help in various ways (Zimet et al., 1988; Halbesleben, 2006; Feeney and Collins, 2015). Social support can be both beneficial in addressing the psychological challenges in people’s lives and can provide direct help in dealing with difficulties (Cohen and Wills, 1985). On the one hand, the positive psychological feeling of support and help from the outside world can make students more satisfied with their studies and reduce their psychological stress (Thoits, 2011). On the other hand, social support has an objective support role for individuals. During the pandemic, social support has been critical in helping people cope with life difficulties as well as mental health issues (Van Bavel et al., 2020). Previous research has also found that social support during the pandemic can play an important role in college students’ resolution of academic troubles and difficulties (Yu and Zhou, 2022).

Emotional intelligence has been shown to influence the acquisition of social support (Kong et al., 2012) and to predict social support (Di Fabio and Kenny, 2015). In addition, emotional intelligence may influence college students’ perceptions of their social support (Lopes et al., 2003), and good emotional intelligence may be more likely to be satisfied with the social support they receive. Several studies have revealed that social support predicts learning motivation (Song et al., 2015; Camacho et al., 2021).

Emotional intelligence may make people better able to deal with emotional aspects and help college students maintain adequate levels of learning motivation. However, social support is particularly important when college students face challenges that are beyond their abilities (Fortes et al., 2022). Social support can help college students to be better equipped and confident in dealing with challenges and difficulties, and thus better able to cope with stress and anxiety in their learning (Zhou and Yu, 2021). Therefore, emotional intelligence may combine with social support to further influence academic motivation. However, whether social support mediates the connection between emotional intelligence and learning motivation during the pandemic needs more exploration.

### Self-efficacy and social support play serial mediation roles between emotional intelligence and learning motivation

In previous research, it was found that self-efficacy and social support can together constitute an important resource for college students to cope with challenges (Li et al., 2018). An important reason for the correlation between self-efficacy and social support may be that they are both related to one’s overall perception of solving difficulties. Self-efficacy is the perception that people believe they can solve problems and deal with challenges, while social support is the overall perception that they can get external support when they encounter difficulties. In addition to their efforts to solve problems, some of the learning challenges faced by college students often require support from external forces to solve them (Wang and Huang, 2022).

During the pandemic, college students may rarely encounter such a challenging environment, and their self-efficacy may make them more certain of their problem-solving abilities, more motivated to face challenges, and confident that they can obtain more external social support to solve specific learning problems (Warshawski, 2022). Greater social support and the ability to mobilize external support from a wide range of sources can contribute to college students’ confidence in solving their learning problems. This may imply that both self-efficacy and social support can contribute to learning motivation.

In previous studies, emotional intelligence can be both predictive of self-efficacy and may be associated with good perceptions of social support (Rostami et al., 2010). Earlier research has indicated that self-efficacy and social support are closely linked (Vekiri and Chronaki, 2008; Yang et al., 2019; Warshawski, 2022), which makes it necessary to integrate their roles within the connection between emotional intelligence and learning motivation. College students’ emotional intelligence may have a positive effect on self-efficacy and perceived social support, which in turn may promote learning motivation. Considering that connections exist among emotional intelligence, learning motivation, self-efficacy, and social support, we must further explore whether self-efficacy and social support play serial mediating roles between emotional intelligence and learning motivation during the pandemic.

### Present research

Previous research has explored the relationship between emotional intelligence and the psychology of learning among college students, for example, finding a relationship between student learning boredom and emotional intelligence during online learning (Chengchen and Dewaele, 2020). Research on the relationship between student’s emotional intelligence and learning motivation has become a topic of interest to practitioners during the pandemic and exploration of these aspects can lead to a better understanding of how to promote college students’ learning motivation in a challenging environment. Previous studies have investigated students’ state levels of emotional intelligence and motivation (Thomas and Heath, 2022), as well as analyzing the relationship between emotional intelligence and learning motivation (Trigueros et al., 2019). However, there has been little research on the mediating role between emotional intelligence and learning motivation during the pandemic, and there is a need to provide more evidence in this regard with the present study.

Previous research has found a correlation between emotional intelligence and self-efficacy (Hen and Goroshit, 2014), and the relationship between self-efficacy and learning motivation has been confirmed (Yüner, 2020). In some previous studies related to educational contexts, it was initially found that emotional intelligence and self-efficacy can synergistically influence the teaching ability of a group of teachers (Răducu and Stănculescu, 2021). However, previous studies have a less in-depth analysis of whether self-efficacy can play a mediating effect between emotional intelligence and learning motivation. During the pandemic, it is necessary to strengthen the research on the role of self-efficacy in the relationship between emotional intelligence and learning motivation.

Previous literature has suggested that learning motivation may be influenced by social resources and personal conditions (Fatima et al., 2018). However, there is little literature that directly links self-efficacy and social support to examine the role of both in influencing learning motivation. Increasing research on the mediating role between emotional intelligence and motivation during the pandemic will help to understand the underlying mechanisms that increase college students’ learning motivation.

In short, there is a need to increase research on the relationship between emotional intelligence and learning motivation among college students during the pandemic and to explore in depth the complex mechanisms underlying these relationships. Although previous studies have provided clues that self-efficacy and social support may play a mediating role between emotional intelligence and motivation (Gharetepeh et al., 2015; El-Sayed et al., 2021), we know less about the relevant mediating models and the series of mediating effects constituted by different mediating variables. Considering that the pandemic is still spreading worldwide, it is likely that the learning and mental health of university students will continue to face challenges (Heumann et al., 2023). Increasing research in this area may identify more effective interventions to promote learning and better exploit the protective function of emotional intelligence (García-Álvarez et al., 2021), providing useful insights into the promotion of motivation among university students. In addition, conducting such research during the pandemic could help add new evidence to the research on the relationship between emotional intelligence and learning, potentially increasing the understanding of some of the complex mechanisms of how emotional intelligence plays a role in learning motivation and providing new information for the psychological optimization of learning among college students.

**Figure 1:**
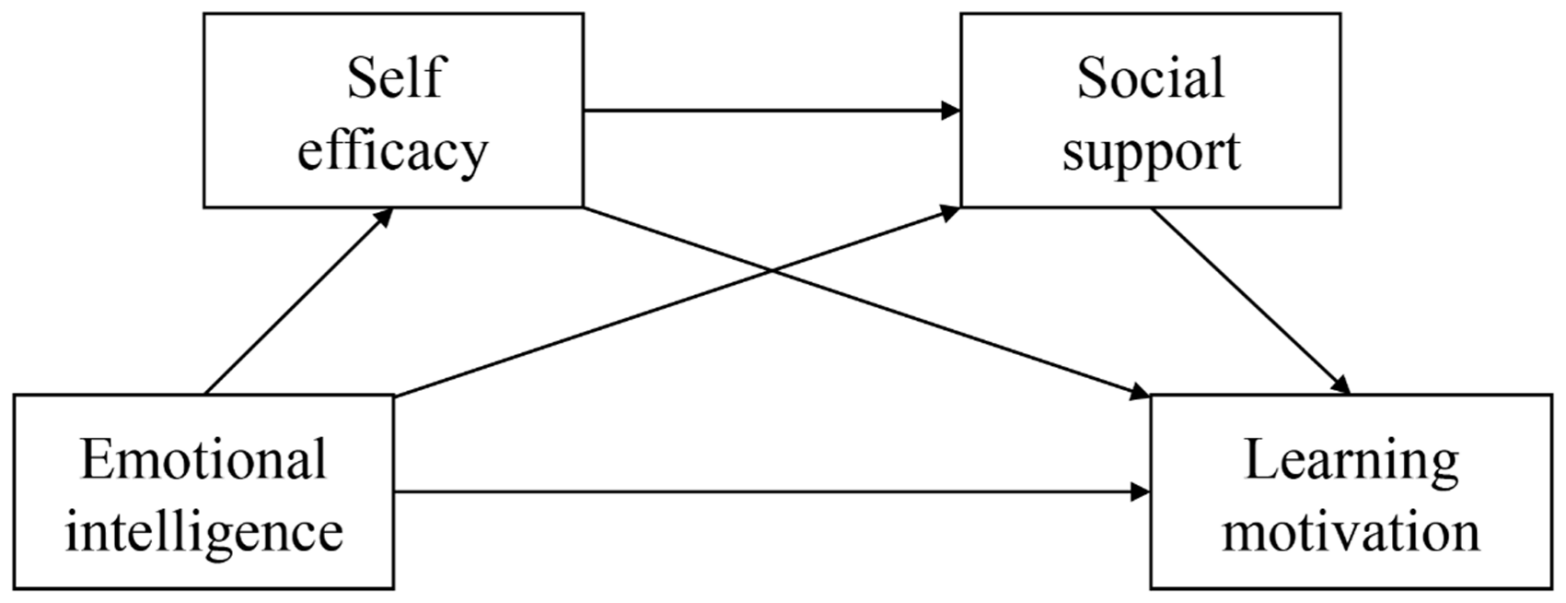
Research hypothesis framework diagram.

Therefore, the current study explored the following questions. What is the correlation among college students’ emotional intelligence and motivation during the COVID-19 pandemic? Can self-efficacy and social support mediate the relationship between emotional intelligence and motivation? We propose the following four hypotheses (See Figure 1).

*H1*: During the COVID-19 pandemic, college students’ emotional intelligence positively predicts learning motivation.

*H2*: During the COVID-19 pandemic, college students’ self-efficacy plays a mediating role between emotional intelligence and learning motivation.

*H3*: During the COVID-19 pandemic, social support among college students mediates the relationship between emotional intelligence and learning motivation.

*H4*: During the COVID-19 pandemic, college students’ self-efficacy and social support play a serial mediating role between emotional intelligence and learning motivation.

## Methods

### Participants and survey process

#### Design

Considering the feasibility of contacting the study participants, we positioned the study population as college students aged eighteen years or older attending Chinese universities. Since it was not easy to conduct an offline survey of college students from different parts of China over the period of the pandemic, we used a convenience sampling method. We used an online platform to recruit college student volunteers, so we had the opportunity to include a wide range of college students from different locations who saw public information on the Internet and volunteered to participate in the survey. We did not limit the participants to specific types of schools, but to avoid over-concentration of participants in a certain area, we used the geographic restriction technology of the participant answering device in the professional online research platform to allow only one participant to answer within a 10-kilometer range so that more college students could participate in the study through popular social media such as WeChat. The basic information reported by the participants showed that they came from many different types of universities, which made the participants representative to a certain extent.

#### Procedure

This investigation was conducted in September 2022, when the normal life of Chinese university students was still affected by the COVID-19 pandemic. The authors’ institution was informed of and consented to the research in advance. At the beginning of the questionnaire, we provided information about the nature of the study, and only when participants provided informed consent could they continue to fill out the questionnaire. Ultimately, we recruited 341 college students from 30 different provinces and over 130 cities in China to complete the questionnaire through a professional questionnaire research online platform. The questionnaire included four scales concerning emotional intelligence, learning motivation, self-efficacy, and social support, in addition to basic demographic information. Participants took a mean of 13 min to fill out the questionnaire. Of the 341 questionnaires, five were discarded because of incomplete information, resulting in a total of 336 questionnaires being adopted, with no missing data in the adopted questionnaires. To achieve sufficient statistical power to test the hypotheses and avoid type I errors, previous studies have suggested that the sample size required for serial mediation model testing generally needs to be greater than 200 (Tofighi and Kelley, 2020). Therefore, the sample size in the present investigation corresponded to this recommendation.

#### Sample characteristics

In this study, there were 336 participants, 77 male (22.92%) and 259 female college students (77.08%). A total of 276 participants were aged 18–22 years old, accounting for 82.14%, 52 participants were aged 23–25 years old, accounting for 15.48%, and 8 participants were aged 26–36 years old, accounting for 2.38%. The participants included undergraduate, master’s, and doctoral students, including 6 doctoral students (1.79%), 59 master’s students (17.56%), and 271 undergraduate students (80.65%) (See Table 1).

**Table 1:** Basic characteristics.

Variable	Category	Frequency	Percentage
Sex	Male	77	22.92%
	Female	259	77.08%
Age	18–22 years	276	82.14%
	23–25 years	52	15.48%
	26–36 years	8	2.38%
Learning level	Doctoral students	6	1.79%
	Master’s students	59	17.56%
	Undergraduates	271	80.65%

### Measures

The emotional intelligence measure was adapted from a questionnaire developed by Schutte et al. (1998), and the Chinese version of this questionnaire was validated (Che et al., 2017; Yang, 2022). This scale contains 33 items and uses a five-point Likert scale, with 30 positively scored questions and 3 reverse scored questions. The questionnaire includes items such as, “I am able to control my emotions.” In this survey, Cronbach’s α value was 0.859, Composite Reliability (CR) was 0.871, and McDonald’s Omega value was 0.888, reflecting that the questionnaire has high reliability. Performing confirmatory factor analysis, the results confirmed the validity of the questionnaires (*χ*^2^/sd = 2.712, RMSEA = 0.071).

Learning motivation was measured using a questionnaire adapted from the Work Preference Inventory scale developed by Amabile et al. (1994). The reliability of the Chinese version of this questionnaire has been demonstrated by previous studies (An et al., 2022). The questionnaire includes 28 positively and 2 negatively scored questions, totaling 30 questions, using a 4-point Likert scale, such as the item “I like to think independently to solve difficult problems.” In this study, Cronbach’s α value was 0.772, the CR value was 0.805, and McDonald’s Omega value was 0.812, reflecting that the questionnaire has high reliability. Performing confirmatory factor analysis, the results confirmed the validity of the questionnaires (*χ*^2^/sd = 3.385, RMSEA = 0.084).

Self-efficacy was assessed using a scale designed by Schwarzer et al. (1999). Preceding research supports the applicability of the Chinese version (Guo et al., 2022; Huang et al., 2022). The questionnaire comprises 10 items, using a 4-point Likert scale. The questionnaire includes items such as, “If I put in the necessary effort, I will be able to solve most of my problems.” The Cronbach’s α value in the current research was 0.861, indicating its reliability. In the present study, the CR value was 0.827, and McDonald’s Omega value was 0.889, reflecting that the questionnaire has high reliability. Performing confirmatory factor analysis, the results confirmed the validity of the questionnaires (*χ*^2^/sd = 3.241, RMSEA = 0.082).

Social support was tested with the scale prepared by Zimet et al. (1990). The good measurement characteristics of the Chinese version have been confirmed (Li et al., 2022; Yin et al., 2022). The 12-item questionnaire uses a seven-point Likert scale, and all items are positively scored. The questionnaire includes items such as, “My friends can really help me.” The Cronbach’s α value was 0.868 in the present investigation, the CR value was 0.862, and McDonald’s Omega value was 0.895, reflecting that the questionnaire has high reliability. Performing confirmatory factor analysis, the results confirmed the validity of the questionnaires (*χ*^2^/sd = 2.904, RMSEA = 0.075).

### Data analysis

The score means and standard deviations were calculated for college students on four scales: emotional intelligence, learning motivation, self-efficacy, and social support. Since the sample size collected is greater than 300, the multivariate normality distribution of the four variables can be estimated using kurtosis and skewness values (Mishra et al., 2019). The correlations between the variables of emotional intelligence, learning motivation, self-efficacy, and social support were analyzed using Pearson’s correlation coefficient. In comparing the differences in college students’ scores in these scales according to gender, age, and level of learning, this study used analysis of the Kruskal-Wallis test and student’s *t*-test. In the regression analysis, the IBM SPSS software, and the micro Process plug-in Model 6 developed by Hayes (2017) were used for mediation analysis. Mediating effects were analyzed using bootstrap tests, setting the number of replications to 5,000. A mediating effect was considered to exist when the upper and lower 95% confidence intervals did not include zero. The proportion of each mediating effect value to the total effect was analyzed.

## Results

Whether the data are normally distributed can be examined by analyzing the skewness and kurtosis of the variable data (Kim, 2013; Orcan, 2020). The skewness and kurtosis of the data of the four variables were calculated and it was found that their kurtosis and skewness absolute values were less than 1. Therefore, the data of the four variables of emotional intelligence, learning motivation, self-efficacy, and social support can be accepted as essentially normally distributed, as shown in Table 2.

**Table 2:** Results of multivariate normality analysis.

Variable	*N*	Kurtosis values	Skewness values
Emotional intelligence	336	−0.115	0.079
Learning motivation	336	−0.017	0.252
Self-efficacy	336	0.115	0.38
Social support	336	0.306	−0.329

The range of scores on the four scales, the mean total scores, and the standard deviation of the total scores were tallied for all college participants, and the correlations among the variables of emotional intelligence, learning motivation, self-efficacy, and social support were analyzed using Pearson’s correlation coefficients (See Table 3). Emotional intelligence was positively correlated with motivation to learn (*r* = 0532, *p* < 0.01), self-efficacy (*r* = 0.548, *p* < 0.01), and social support (*r* = 0.536, *p* < 0.01). Learning motivation was positively associated with both self-efficacy (*r* = 0.406, *p* < 0.01) and social support (*r* = 0.394, *p* < 0.01). Self-efficacy and social support were also positively correlated (*r* = 0.392, *p* < 0.01).

**Table 3:** Range, mean score, standard deviation, and correlation between variables for each scale score.

Variable	Range	Mean	Standard deviation	Emotional intelligence	Learning motivation	Self-efficacy	Social support
Emotional intelligence	99 ~ 158	126.99	11.38	1			
Learning motivation	67 ~ 114	89.42	7.88	0.532**	1		
Self-efficacy	16 ~ 40	27.00	4.45	0.548**	0.406**	1	
Social support	25–83	62.77	9.38	0.536**	0.394**	0.392**	1

^**^p < 0.01.

Differences between groups were compared for the four variables according to gender. The difference between the means of the two groups was analyzed by Student’s *t*-test and the effect size of the difference was measured by using Hedge’s g. It can be found that the scores between self-efficacy and learning motivation showed significant differences between the different gender groups, and this difference was a small effect (See Table 4).

**Table 4:** Differences between the different gender groups.

	Male	Female	*T*	Effect size
Emotional intelligence	127.91 ± 10.97	126.72 ± 11.51	0.8	0.117
Learning motivation	91.10 ± 7.86	88.92 ± 7.83	2.15^*^	0.278
Self-efficacy	28.12 ± 4.75	26.67 ± 4.31	2.53^*^	0.328
Social support	62.70 ± 8.51	62.80 ± 9.64	−0.08	−0.011

*p < 0.05.

The Kruskal-Wallis test was used to analyze the differences between the age groups and found that the three variables of learning motivation, self-efficacy, and social support did not show significant differences, but emotional intelligence showed significant differences (See Table 5). The Least Significant Difference (LSD) Test was conducted on the emotional intelligence of different age groups and found that the mean score of the 26–36 age group was significantly higher than that of the 18–22 age group (*p* = 0.020).

**Table 5:** Differences between the different age groups.

	Median M (P_25_, P_75_)			*H*	*p*	Effect size
	18–22 years (*n* = 276)	23–25 years (*n* = 52)	26–36 years (*n* = 8)			
Emotional intelligence	126.0 (119.0, 134.0)	127.5 (123.0, 136.8)	131.5 (129.3, 146.5)	6.039	0.049*	0.021
Learning motivation	89.0 (84.0, 95.0)	88.5 (86.0, 92.8)	92.0 (85.8, 105.5)	1.92	0.383	0.011
Self-efficacy	26.5 (24.0, 30.0)	26.0 (24.3, 29.0)	30.0 (26.3, 36.8)	4.458	0.108	0.021
Social support	62.0 (56.0, 69.0)	65.5 (56.5, 73.8)	70.5 (60.3, 78.0)	5.805	0.055	0.019

*p < 0.05.

Analysis using the Kruskal-Wallis test reveals that between the different learning level groups, emotional intelligence and motivation scores did not show significant differences, but self-efficacy and social support showed significant differences. Using partial eta squared to calculate the differential effect sizes, it was found that the differential effect sizes between self-efficacy and social support between different learning levels were small (See Table 6). Calculations using the Least Significant Difference (LSD) Test method revealed that the mean self-efficacy scores of doctoral students were significantly higher than those of the undergraduate group (*p* = 0.001), and the mean self-efficacy scores of doctoral students were significantly higher than those of the master’s group (*p* = 0.003). In terms of social support, the master’s student group was significantly higher than the undergraduate student group (*p* = 0.005).

**Table 6:** Differences between different learning level groups.

	Median M (P25, P75)			*H*	*p*	Effect size
	Undergraduates (*n* = 271)	Master students (*n* = 59)	Doctoral students (*n* = 6)			
Emotional intelligence	126.0 (119.0, 135.0)	127.0 (123.0, 135.0)	131.5 (128.5, 152.3)	5.207	0.074	0.022
Learning motivation	89.0 (84.0, 95.0)	89.0 (86.0, 94.0)	92.0 (87.3, 107.3)	2.818	0.244	0.015
Self-efficacy	26.0 (24.0, 30.0)	27.0 (25.0, 30.0)	32.5 (27.5, 38.5)	6.854	0.032*	0.032
Social support	62.0 (55.0, 69.0)	66.0 (59.0, 71.0)	72.5 (56.3, 79.3)	8.963	0.011*	0.030

*p < 0.05.

**Figure 2:**
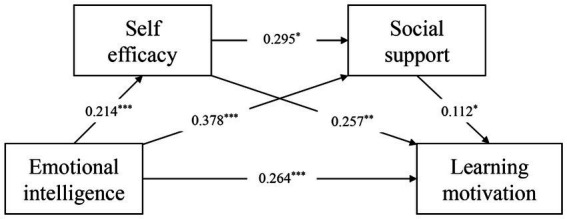
Analysis of the coefficients of the mediated pathways. *p < 0.05, **p < 0.01, ***p < 0.001.

To test the mediating effects model proposed in the study, we analyzed the significance of the coefficients in the linear regression analysis. Regression analysis yielded a positive predictive effect of college students’ emotional intelligence on college students’ self-efficacy (*B* = 0.214, *t* = 11.985, *p* < 0.001). Regression analysis also found that college students’ emotional intelligence (*B* = 0.264, *t* = 6.376, *p* < 0.001), self-efficacy (*B* = 0.257, *t* = 2.640, *p* < 0.001), and social support (*B* = 0.112, *t* = 2.446, *p* < 0.05) positively predicted college students’ learning motivation. This reflects the fact that self-efficacy and social support play a series of mediating roles in emotional intelligence and learning motivation. In this model, emotional intelligence, self-efficacy, and social support explained 31.4% of learning motivation (*F* = 50.706, *p* < 0.001). The VIF values and D-W values in the linear regression equations involved in this mediated model were calculated separately, and it was found that the VIF values in the model were all less than 5, indicating that there was no covariance problem. And the D-W values are all around 2, reflecting the absence of autocorrelation in the model, which indicates that the model is well constructed (See Table 7). As a result, a mediation model including path coefficients could be obtained (See Figure 2).

**Table 7:** Regression analysis results of the mediation model.

Dependent variable	Independent variable	*B*	*t*	*p*	95% Confidence interval	VIF	*R* ^2^	*F*	*p*	D-W
Self-efficacy							0.3	144	*p* < 0.001	1.73
	Emotional intelligence	0.2	12	*p* < 0.001	[0.1792, 0.2495]	1				
Social support							0.3	71.7	*p* < 0.001	2.13
	Emotional intelligence	0.4	8.4	*p* < 0.001	[0.2896, 0.4672]	1.43				
	Self-efficacy	0.3	2.6	*p* < 0.05	[0.0678, 0.5221]	1.43				
Learning motivation							0.3	50.7	*p* < 0.001	1.76
	Emotional intelligence	0.3	6.4	*p* < 0.001	[0.1826, 0.3456]	1.73				
	Self-efficacy	0.3	2.6	*p* < 0.01	[0.0654, 0.4480]	1.46				
	Social support	0.1	2.4	*p* < 0.05	[0.0219, 0.2017]	1.43				

Findings from the mediation analysis indicated that self-efficacy among college students played a significant mediating role between emotional intelligence and motivation, with an effect value of 0.0550, 95% CI [0.0127, 0.1006]; the amount of this path effect as a percentage of the total effect was 14.93%. Mediation of social support between emotional intelligence and motivation was significant, with an effect value of 0.0423, 95% CI [0.0051, 0.0823], and the proportion of this pathway effect was 11.48% of the total effect. Self-efficacy and social support exerted significant serial mediation function between emotional intelligence and motivation, with an effect value of 0.0071, 95% CI [0.0003, 0.0176], and the proportion of this pathway effect was 1.93% of the total effect (See Table 8). The calculation of the mediating effect reveals that the direct effect is the strongest statistical path in the model, with an effect size of 71.67% of the total effect, while the indirect effect accounts for 28.33% of the total effect. Among the indirect effects, self-efficacy played the most significant effect in mediating between emotional intelligence and learning motivation.

**Table 8:** Analysis of intermediary effects.

Pathway	Effect size	BootSE	95% Confidence interval(CI)	Ratio of effect size to total effect
Direct pathway	0.2641	0.0414	[0.1826, 0.3456]	71.67%
Emotional intelligence→Learning motivation				
Indirect pathways	0.1044	0.0303	[0.0474, 0.1655]	28.33%
Ind1:Emotional intelligence→Self efficacy→Learning motivation	0.0550	0.0229	[0.0127, 0.1006]	14.93%
Ind2:Emotional intelligence→Social support→Learning motivation	0.0423	0.0195	[0.0051, 0.0823]	11.48%
Ind3: Emotional intelligence→Self efficacy→Social support→Learning motivation	0.0071	0.0045	[0.0003, 0.0176]	1.93%
Ind1 − Ind2	0.0127	0.0315	[−0.0475, 0.0753]	/
Ind1 − Ind3	0.0480	0.0237	[0.0033, 0.0945]	/
Ind2 – Ind3	0.0352	0.0174	[0.0043, 0.0726]	/

## Discussion

Encouragingly, we found that emotional intelligence positively predicted learning motivation and that self-efficacy and social support played serial mediating roles between emotional intelligence and motivation during the COVID-19 pandemic. We found that older college students scored higher on emotional intelligence compared to younger college students. This is consistent with previous studies (Naidoo, 2008; Todres et al., 2010) and maybe because older undergraduates are more physically mature and have more life and social experience, which helps them to better identify and control their emotions. In terms of self-efficacy, the doctoral group scored higher than the other learning level groups, which may be due to the stronger academic achievements of doctoral students compared to students at other levels, which contributes to their affirmation of self-efficacy. This finding further supports previous research (Cheng et al., 2019). In terms of social support, master’s students were more able to appreciate the support from those around them compared to undergraduate students, consistent with previous research (Hefner and Eisenberg, 2009). This may be because master’s students are educated in a different way than undergraduate students. Master’s degree students are usually supervised by a dedicated supervisor, have a closer student-teacher relationship, are better supported in their studies by their supervisory team, and thus may perceive more social support.

The findings suggest that emotional intelligence positively predicts learning motivation, supporting Research Hypothesis 1. This study confirmed previous findings (Yenice et al., 2018; Rodriguez-Gonzalez et al., 2021). Emotional intelligence is an essential psychological ability, which supports and influences college students’ learning (AL-Qadri and Zhao, 2021). Students need a certain level of emotional regulation capabilities if they are to maintain good motivation. Emotional intelligence enables college students to better recognize, control, and regulate their emotions; this allows them to experience more positive emotions and increases their interest in learning, thereby increasing their motivation to study. Emotional intelligence can help students overcome negative emotions associated with academic difficulties and perform more positively academically (Brackett et al., 2011), thus maintaining enthusiasm to participate in learning.

College students may face many learning obstacles, and emotional intelligence can help students develop a sense of self-awareness to face these difficulties and develop a good learning mindset and habits (Iqbal et al., 2022)—thus enabling them to maintain sufficient enthusiasm and commitment in their studies. During the pandemic, college students may face more difficulties in their academic studies. These difficulties and challenges make it more likely that they will face emotional distress. Globally, the increased stress in the curriculum, the disruption of the rhythm of life, the disruption of social life, and the risk of being infected by the virus due to the disruption of normal studies can be negative factors that affect the learning motivation of university students (Marler et al., 2021). In particular, college students have to face changes in their learning style during the pandemic, which can affect their learning status at a deeper level. Emotional intelligence can bring more positive psychological strength to college students, making them more optimistic and positive in coping with learning difficulties, which is quite important for the protection of college students’ learning motivation during the pandemic (Rahiem, 2021). In addition, emotional intelligence can help college students to better manage life and interpersonal tasks, overcome challenges in life and social interactions (Nasir and Masrur, 2010), and provide them with the mental resources and focus to better carry out their studies. Emotional intelligence may directly or indirectly help college students to overcome these learning difficulties by helping them to deal with related emotional challenges, thus ensuring their learning motivation.

We found that college students’ self-efficacy mediated the relationship between emotional intelligence and learning motivation, supporting Research Hypothesis 2. Our study confirms the existence of found a relationship between self-efficacy and emotional intelligence, as reported by previous studies (Salavera et al., 2017; Wen et al., 2020). Emotional intelligence avoids emotional exhaustion by increasing positive emotional experiences and decreasing negative emotional experiences, making it easier for people to overcome adversity, gradually accumulate positive psychological resources, and increase confidence in their learning and work, which in turn contributes to the formation of self-efficacy (Gharetepeh et al., 2015). University students who have higher emotional intelligence may be patient in solving difficulties in learning, thus actively participating in learning and contributing to their good self-efficacy (Bonilla-Yucailla et al., 2022). An increase in self-efficacy can broadly affect people’s mental state, enhance self-satisfaction and self-confidence, and make people more goal-oriented, which may have a motivating effect on students’ learning on multiple levels (Skinner et al., 2022). Thus, emotional intelligence can be an enabling factor for increased self-efficacy, and students with good self-efficacy are likely to adopt more effective learning strategies, which may also provide the basis for their increased learning motivation.

Our findings further support previous studies linking self-efficacy to learning motivation (Khoso et al., 2022). Self-efficacy helps students overcome difficulties more positively and serves to protect their psychological wellness (Raskauskas et al., 2015). College students may face very many challenges during the pandemic, for example, the negative effects of online learning and exams may also affect their perception of the meaning of learning (Daniels et al., 2021). In this challenging environment, they need more positive psychological forces to solve problems, manage to learn, and motivate learning. The self-efficacy of college students motivates them to be more active in finding ways to adapt to the new environment (Zeng et al., 2021). This makes them more confident in coping with the risks posed by the pandemic and more willing to stay engaged in their studies. College students with higher self-efficacy can be more motivated to handle challenges, which would help with avoiding frustration and maintaining higher levels of motivation in their studies. Therefore, a conscious effort to develop students’ self-efficacy during the pandemic is necessary.

The mediating role of social support between emotional intelligence and learning motivation was verified, supporting Research Hypothesis 3. This study confirms earlier findings (Shuo et al., 2022), and further supports the idea that college students who have good social support may have better learning motivation (Wentzel et al., 2010; Marley and Wilcox, 2022). Different individuals may feel differently under similar conditions of social support. Good emotional intelligence may help people to more clearly appreciate the external support they may receive (Metaj-Macula, 2017) and be more likely to feel emotionally fulfilled. Emotional intelligence can play a facilitating role in active support perceptions, which may further influence people’s behavioral intentions, such as promoting college students’ learning motivation. For college students during the pandemic, perceiving social support from those around them is quite important and helps them maintain positive psychology in the face of difficulties (Guo et al., 2021), thus being more motivated to continuously engage in their studies.

During the pandemic, college students need to rely on family, school, or friends to overcome their difficulties, in addition to emotional regulation to increase their confidence and perseverance overcome their difficulties (Lederer et al., 2021). Although college students’ emotional intelligence can help them better deal with emotional problems, they require further external learning, psychological interventions, or financial support during the COVID-19 pandemic (Akbar and Aisyawati, 2021; Tozini and Castiello-Gutierrez, 2022). College students who lack social support often experience serious mental health problems (Yin et al., 2021).

It can be inferred that social support plays an important role in facilitating the relationship between emotional intelligence and learning motivation. On the one hand, with emotional intelligence, college students may have more social skills (Trigueros et al., 2020), have better interpersonal relationships (Mercader-Rubio et al., 2022b), be better able to communicate and interact with society and receive more external support. Emotional intelligence can help college students to better access social support (Barros and Sacau-Fontenla, 2021) and thus motivate them to engage seriously in their studies. On the other hand, social support helps college students adopt appropriate ways to cope with their academic difficulties and provides them with emotional support at the psychological level. Social support may help college students employ effective coping strategies to resolve difficulties (Mai et al., 2021) as well as develop good social–emotional perceptions—an emotional state that contributes to motivation (Latorre-Cosculluela et al., 2022). Students who perceive that they receive good social support are more motivated to stay on the learning track and perform well in school (Fernandez-Lasarte et al., 2019). For this reason, it is important to focus on providing adequate social support for college students during the pandemic.

This study suggests that college students’ self-efficacy and social support acted as serial mediators between college students’ emotional intelligence and learning motivation, supporting Research Hypothesis 4. A correlation between self-efficacy and social support has already been found in previous studies (Yenen and Carkit, 2021). Previous studies have also discovered a correlation between self-efficacy and learning motivation (McGeown et al., 2014; Salazar and Hayward, 2018; Yüner, 2020), and social support has been found to be relevant to learning motivation (Emadpoor et al., 2016; Klootwijk et al., 2021). Our study findings correspond with the aforementioned reports. Interestingly, self-efficacy and learning motivation can function by acting as chain mediators between emotional intelligence and learning motivation.

Emotional intelligence has the potential to enable college students to have good emotional regulation and help facilitate the functioning of other types of positive psychological resources (da Costa et al., 2021). The self-efficacy and social support of college students may be better supported by emotional intelligence as a facilitator of learning. The college students’ self-efficacy represents an internal psychological resource (Schreitmueller and Loerbroks, 2020) that is related to individuals’ perceptions of their own abilities, whereas social support is related to external help (Ferber et al., 2022). Self-efficacy and social support represent internal psychological strengths and external support resources, respectively, that can provide support for college student’s mental health and learning. Self-efficacy and social support enable college students to actively engage in their studies when they face learning difficulties (Rigg et al., 2013). The combination of these two could better help college students cope with difficulties in school and life. This would imply that college students can regulate their negative emotions with support from their emotional intelligence (Moron and Biolik-Moron, 2021), and gradually increase their self-efficacy, thus becoming more motivated to seek external support and help as well as engage in their studies with confidence and enthusiasm. During the pandemic, the positive effects of emotional intelligence on learning can be better reflected with the help of self-efficacy and social support facilitation, thus helping college students to increase their motivation to learn. Self-efficacy can motivate college students to face difficulties, and it can also enhance their ability to access external resources (Talsma et al., 2021). College students also need adequate external support during the pandemic to better survive their studies. Thus, it can be predicted that emotional intelligence, self-efficacy, and social support are protective factors for college students’ learning, which can reduce their burnout, help them better overcome their learning difficulties, increase their confidence and commitment to learning, and thus enhance their motivation. These findings inspire us to better integrate internal self-efficacy training and external social support to enable college students to use their emotional intelligence for promoting learning motivation.

### Main contributions

Promoting student motivation and enhancing student performance during the COVID-19 pandemic is a global issue, but the underlying mechanisms behind this have not been clarified. This study has three main research contributions. First, it found that college students’ emotional intelligence positively predicted their learning motivation, thereby further expanding the research in this field. Second, it explored possible mechanisms between college students’ emotional intelligence and learning motivation and identified evidence that both self-efficacy and social support mediate the connection between college students’ emotional intelligence and learning motivation. Ultimately, it found that college students’ self-efficacy and learning motivation can play serial mediation functions between emotional intelligence and learning motivation; this finding can help educators to better utilize college students’ emotional intelligence for promoting motivation. These findings make a practical contribution to how emotional intelligence can be used to promote motivation during the pandemic and thus enhance college students’ academic performance. This research provides a clearer understanding of the need for systematic interventions to promote college students’ motivation, not only by focusing on the improvement of their internal emotional intelligence and self-efficacy but also by providing them with the support they need to maintain high levels of motivation during the pandemic. This finding may avoid the misconception that relying solely on psychological counseling can solve college students’ learning problems and inspire the need to also focus on providing broad social support. This also further extends previous research and provides new information for studies that promote learning motivation among college students.

### Practical implications

This research provides useful insights for practitioners. First, emotional intelligence is closely related to learning motivation, and the fundamental role of emotional intelligence in learning motivation implies that schools should attach more weight to cultivating college students’ emotional intelligence. Second, promoting university students’ self-efficacy through classroom training and psychological interventions can enhance the influence of emotional intelligence on motivation. Third, social support can both help college students overcome risk challenges as well as facilitate the associations between emotional intelligence and learning motivation. More social support should be provided to college students to enhance their motivation. Fourth, both self-efficacy and social support can function as mediators within the connection between emotional intelligence and learning motivation. People have to attach importance to cultivating university students’ self-efficacy and providing college students with sufficient social support. By encouraging college students to enhance both self-efficacy and social support, emotional intelligence can be better utilized to promote college students’ learning motivation. For this reason, educators should work to foster students’ emotional intelligence to promote motivation.

### Limitations and future direction

The current study has several limitations. First, this investigation mainly utilized a convenience sampling approach. This sampling approach may have a limitation in that self-selection effects may have biased the results (Andrade, 2020), which may mean the results need further validation to enable generalization. Second, the volunteers in the present investigation were recruited in China, which means that the generalizability of the current study’s results to other countries requires additional investigation as well as analysis in multiple countries simultaneously. Third, this study adopted a cross-sectional investigation design, which prevented this study from drawing causal conclusions, and future studies could use longitudinal designs to strengthen the findings. Fourth, this study measured the relevant variables by means of college students’ self-report, and social desirability bias could have affected the accuracy of the results, which may make it difficult to generalize results from the present research to a wider group. The findings derived from the present research can be more comprehensively verified in the future by combining multiple measures. Fifth, although this study found that self-efficacy and social support can play a series of mediating roles in emotional intelligence and learning motivation, the indirect effect value was low. This indirect effect could be further tested in future studies on different groups of university students from different countries. In addition, there may be other mediating pathways with stronger indirect effects that need to be further explored. Sixth, the high proportion of female participants in this study may cause some bias in the results, and the proportion of male and female participants can be controlled appropriately in future studies to make the results more reliable.

## Conclusion

By examining the correlations between Chinese college students’ emotional intelligence and motivation during the COVID-19 pandemic, we found that emotional intelligence positively predicted college students’ learning motivation. Furthermore, self-efficacy and social support were mediators of the connection between emotional intelligence and learning motivation, and self-efficacy and social support acted as serial mediators of the relationship between emotional intelligence and learning motivation. The current research innovatively investigates the connection and potential mechanisms between college students’ emotional intelligence and learning motivation during the COVID-19 pandemic, and expands existing research regarding the significant contribution of emotional intelligence toward education. Our study findings can act as a reference for policymakers and practitioners to better implement emotional intelligence interventions and promote academic performance. Given that the COVID-19 pandemic is not yet over, the potential mechanisms of the impact of emotional intelligence on education throughout the pandemic should be further investigated.

## Data availability statement

The raw data supporting the conclusions of this article will be made available by the authors, without undue reservation.

## Ethics statement

The studies involving human participants were reviewed and approved by Academic Committee of School of Marxism (College of Social Sciences) of Shenzhen University. The patients/participants provided their written informed consent to participate in this study.

## Author contributions

YT: methodology, funding acquisition, and writing—original draft preparation. WH: formal analysis, writing—review and editing, and project administration. YT and WH: investigation. All authors have read and agreed to the published version of the manuscript.

## Funding

This research was funded by the Ministry of Education Teachers of Ideological and Political Theory in Colleges and Universities Special Program, “Research on the Integration of ‘Four Histories’ Education into the Teaching of Ideological and Political Courses in Colleges and Universities during the Intelligent Era” (Funder: Ministry of Education of China; Funding number: 21JDSZK092), and the Shenzhen Philosophy and Social Science planning project, “Embodied Communication of Red Culture among College Students in the Guangdong-Hong Kong-Macao Greater Bay Area in the Metaverse Era” (Funder: Shenzhen Academy of Social Sciences; Funding number: SZ2022C001), and Guangdong Provincial Education Science Planning Project in 2022 (Higher Education Special Project), “Research on the Model and Strategy of High Quality Development of Higher Education in Guangdong Province from the Perspective of Innovation Ecosystem”(Funder: Office of the Leading Group of Education Science Planning of Guangdong Province; Funding number: 2022GXJK077).

## Conflict of interest

The authors declare that the research was conducted in the absence of any commercial or financial relationships that could be construed as a potential conflict of interest.

## Publisher’s note

All claims expressed in this article are solely those of the authors and do not necessarily represent those of their affiliated organizations, or those of the publisher, the editors and the reviewers. Any product that may be evaluated in this article, or claim that may be made by its manufacturer, is not guaranteed or endorsed by the publisher.
